# The Influence of Zinc Oxide and Zinc Stearate on the Antimicrobial Activity of Coatings Containing Raspberry and Chokeberry Extracts

**DOI:** 10.3390/molecules29153493

**Published:** 2024-07-25

**Authors:** Małgorzata Mizielińska, Artur Bartkowiak

**Affiliations:** Center of Bioimmobilisation and Innovative Packaging Materials, Faculty of Food Sciences and Fisheries, West Pomeranian University of Technology Szczecin, Janickiego 35, 71-270 Szczecin, Poland; malgorzata.mizielinska@zut.edu.pl

**Keywords:** antimicrobial coatings, plant extracts, CO_2_ plant extracts, zinc oxide, zinc stearate

## Abstract

The goal of this research was to analyse the synergistic effect between selected plant extracts with zinc oxide particles, and zinc stearate. The influence of ZnO on the antimicrobial effectiveness of the selected extracts was confirmed in previous research carried out by the authors. However, the impact of zinc stearate on extract activity has yet to be analysed. The aim was to cover PLA films with active coatings based on hydroxy-propyl-methyl-cellulose (HPMC), or/and ethyl cellulose (EC) containing plant extracts and ZnO which has a synergistic effect. An additional aim was to use a CO_2_ extract of raspberry seed (RSE) with zinc stearate as active additives within the coatings. An examination of the antimicrobial properties (against *Staphylococcus aureus*, *Escherichia coli*, *Bacillus subtilis*, *Pseudomonas syringae* and Φ6 bacteriophage) of the covered films, as well as an investigation of layer presence with regards to PLA morphology (SEM, ATR-FTIR analysis) was carried out. The research work that was performed indicated that black chokeberry extract (ChE) and zinc oxide particles were effective against *S. aureus*, *P. syringae* and *B. subtilis* strains. In addition, the ChE with zinc stearate (ZnSt) was active against all analysed strains. The HPMC with ChE and ZnO as additives had antimicrobial properties against *S. aureus*, *P. syringae* and *E. coli* strains. The ChE was found to inhibit the growth of all of the analysed bacterial strains. When considering the coatings based on EC with the CO_2_ extract of raspberry seed (RSE) and ZnO, it was noted that they were only active against Gram-negative bacteria. The results of the experiments confirmed that AC1 (EC with RSE with ZnO) and AC2 (EC with RSE with ZnSt) coatings were not active against a phi6 bacteriophage. The HPMC coating containing the AC3 layer (ChE and ZnO) eliminated Φ6 particles, confirming its antiviral properties. In addition, the presence of the active (AC1, AC2 and AC3) coatings was confirmed by SEM and FTIR analysis.

## 1. Introduction

An accelerated increase in bacterial resistance to antibiotics has led to an ever-in-creasing death rate for thousands each year. The growing number of bacterial strains which are resistant to commonly used antibiotics is a very serious problem, even for drugs of last resort, such as vancomycin. The speed with which resistance genes may spread around the world is confirmed by the decrease/fall in the possibility of bacterial disease treatment [1,2]. A dynamic covalent polymeric antimicrobial agent based on phenyl-boronic acid (PBA)-installed micellar nanocarriers incorporating clinical vancomycin and curcumin may be developed to overcome drug-resistant bacterial infections in human treatment. However, it is not only humans that are infected by bacteria, but also food products contaminated by pathogenic strains leading to problems of severe impurities in food, food processing and other areas which can also directly affect human health [3]. To avoid the growth of microorganisms on food products, spoilage processes and the spread of resistance genes, manufacturers add chemical food preservatives. However, researchers are investigating natural preservation methods, including the addition of active substances, such as plant extracts which are rich in bioactive compounds (e.g., saponins, polyphenols and iridoids) [3,4]. Natural compounds extracted from plants have been confirmed to inhibit bacterial pathogens. Among many fruit berries, *Aronia (Aronia melanocarpa*, black chokeberry) is known to have an antibacterial effect on Gram-positive and Gram-negative bacteria [5]. The fruits of the chokeberry are rich in vitamins and minerals. They are also rich in polyphenols, such as anthocyanins, flavonoids and phenolic acids. *A. melanocarpa* anthocyanins are mainly composed of four components, cyanidin-3-glucoside, cyanidin-3-galactoside, cyanidin-3-xyloside and cyanidin-3-arabinoside [6,7]. Active compounds extracted from *A. melanocarpa* may damage the cell wall, resulting in the leakage of intracellular material. These substances may increase ROS (reactive oxygen species) generation and induce oxidative stress in the cells of microorganisms. They can also do the following: 1. influence protein biosynthesis; 2. inhibit ATP synthesis; 3. suppress DNA synthesis. Polyphenols as active agents may have an influence on biofilm formation by affecting the quorum sensing mechanism or pigment production. Additionally, at higher concentrations, polyphenols may inhibit the growth of bacteria; however, at lower concentrations these substances can stimulate bacterial cell growth [8]. It should be mentioned that the ethanol extract of chokeberry was confirmed to be effective against *Bacillus cereus*, *Staphylococcus aureus*, and *Salmonella* Enteritidis [9], *S. enterica*, *Escherichia coli*, *Listeria monocytogenes*, *Pseudomonas aeruginosa* and *Candida albicans* [10]. However, the extract was seen to be more active against Gram-positive rather than Gram-negative bacteria [9]. Due to high antimicrobial activity, the ethanol extract of chokeberry (its water solution, after ethanol evaporation), may be added to a hydrophilic coating carrier, such as HPMC to obtain effective, active layers.

**Figure 1:**
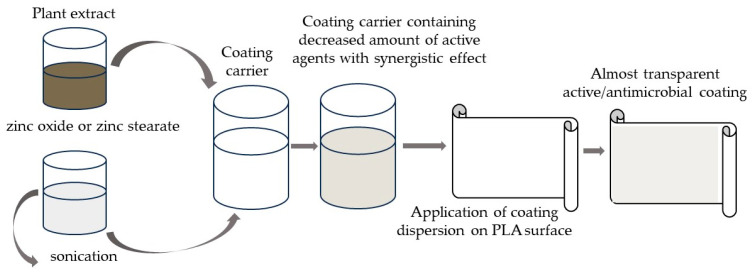
The preparation of active coating containing active agents with synergistic effect.

Supercritical extraction enables researchers to obtain highly active extracts which may not only reduce, but can even inhibit, the growth of many bacterial, yeast and mould strains [10]. In addition, supercritical CO_2_ technology is an environmentally friendly method [11]. Raspberry CO_2_ extract was found to contain highly active compounds, such as anthocyanins, polyphenols, flavanols, hydroxycinnamic acids, hydroxybenzoic acids and hydroxybenzoic acids. Furthermore, the known antibacterial mechanisms of action of this active agent are efflux pump inhibition or the disruption of the bacterial plasma membrane. These action mechanisms allow an extract of raspberries to demonstrate antibacterial, antifungal and also antiviral properties [10,11,12]. In addition, due to its high antimicrobial effectiveness, CO_2_ hydrophobic extract of raspberry seed can be added to the carrier’s hydrophobic coating, such as ethyl cellulose to create layers with antimicrobial properties. Many researchers have suggested that due to its high antibacterial and antioxidant activity, chokeberry extract or CO_2_ extract of raspberry seed could be used as a natural food additive, rather than chemical preservatives. It could be utilized as a naturally derived additive for maintaining the safety of various food products and to avoid food deterioration [5,6,7,8,9,13]. Additionally, to avoid food spoilage caused by bacteria and to limit the use of preservatives, packaging materials could be recommended, especially in the case of those dedicated for short-term stored food products. From this perspective, eco-friendly biopolymers which are biodegradable are seen as promising packaging materials. Among biopolymers, aliphatic polyesters, such as poly(lactic acid) (PLA) appear to be a good replacement for synthetic, non-biodegradable polymers [14,15,16]. PLA has many advantages for packaging applications, being transparent, non-toxic and biodegradable. It may be used as packaging for short-life food products which do not require a high water vapour barrier or a high barrier towards gases [16]. Nevertheless, to make PLA more attractive as a packaging material in terms of food product preservation, the coating process with antimicrobial active layers is a promising alternative [14,15,16]. Active coatings with antimicrobial properties which cover surfaces of biopolymer or polymer films to create protective layers are mostly prepared based on biopolymers, such as polysaccharides. The addition of active agents, e.g., ethanol or CO_2_ plant extracts, into polysaccharides (as coating carriers) has led to the creation of antimicrobially effective coatings. Hydroxy-propyl-methyl cellulose (HPMC) is a natural, partly O-methylated and O-(2-hydroxy-propylated) cellulose derivative. Due to the lower amount of hydrophobic functional groups, the polymer is highly water soluble [17,18]. However, ethyl cellulose, which is a cellulose derivative, is one of the most important hydrophobic polysaccharides. It is considered a significant, natural coating carrier (due to its good film-forming properties), and is a biodegradable biopolymer with excellent water resistance (EC is water insoluble), good biological compatibility and high mechanical strength [19]. EC presents several remarkable features, such as thermoplasticity, innocuousness and robustness, good mechanical properties and thermal stability. This biopolymer has been confirmed as an emulsifier, microencapsulating agent and stabilizer that makes it a good coating carrier for antimicrobial coating application [20,21]. From a consumers’ point of view, it is important to obtain biodegradable, active packaging containing natural antimicrobials which are also almost transparent. However, a high concentration of plant extract or solutions of extracts which are dark green or even dark brown in hue may lead to the creation of a non-transparent coating. On the other hand, the addition of a hydrophilic, active agent into the hydrophobic coating carrier with an emulsifier or the introduction of hydrophobic active compounds into a hydrophilic coating carrier with an emulsifier can result in emulsions which create non-transparent, white coatings. It should also be mentioned that the transparency of active layers depends on layer grammage or the amount of active compounds (plant extracts) in the coating carrier [10,22,23,24]. The modification of coating dispersions through a decrease in the amount of active agents (e.g., plant extracts) can lead to the creation of transparent, though inactive, coatings. To avoid this problem, a synergistic effect between active substances might be considered. Excellent examples are results from the authors’ previous research [24], which confirmed that the addition of zinc oxide particles into a coating carrier containing geraniol or carvacrol led to the creation of an antibacterial coating, confirming a synergistic effect between the two active agents (a decreased amount of geraniol or carvacrol in the coating carrier created a nonactive layer, and in addition a decreased amount of ZnO also created a nonactive layer; however, a decreased amount of geraniol or carvacrol with the addition of a decreased amount of ZnO particles led to the creation of active layers). Based on these considerations it was decided that to obtain an active, transparent coating containing a chokeberry extract (which is dark brown) or a raspberry seed extract (which is yellow), the amount of the plant extract should be as low as possible and therefore would result in a non-active coating. That is why the addition of ZnO particles was considered as an effective alternative to increase the effectiveness of the plant extracts. Mohammadipour-Nodoushan et al. reported [25] that zinc oxide particles at the same concentration had the effect of bactericidal cell toxicity, as well as being non-toxic for human cells. Additionally, Zn^2+^ was seen as highly attractive as the loaded ion is particularly capable of cell wall rupture and cytoplasmic leakage to kill bacteria [26]. Due to their ability to remove ions, these particles were found to have a higher toxic effect on bacterial cells than other metal oxide particles, such as TiO_2_ [25]. ZnO particles demonstrated high stability under UV treatment. Additionally, the use of ZnO particles may improve the UV-shielding properties of any active layer applied to packaging materials. The zinc oxide particles can not only protect their own antimicrobial activity, but can even be effective as natural antimicrobial agents [22,23,24,27]. Although ZnO is not toxic to humans, as it is listed as a generally recognized as safe (GRAS) material by the U.S. Food and Drug Administration [28,29,30,31], its amount (as an active agent in the coating) could also be decreased [27]. Mania et al. [28] stated that ZnO particles can be used to obtain active packaging materials as an alternative to antibiotics without leading to the emergence of microorganism drug resistance [9]. It was mentioned [28], that zinc stearate may also be used as an active agent due to its antimicrobial properties, and also hypothesised that the use of zinc ions with different chemical natures—hydrophilic zinc oxide and hydrophobic zinc stearate—can cause a synergistic effect which can lead to greater antimicrobial activity. According to this hypothesis, it was assumed that not only zinc oxide, but also zinc stearate, can be added to the plant extracts to increase effectiveness. It should be stressed that zinc stearate has yet to be used as an active additive to increase the antimicrobial effectiveness of plant extracts (such as chokeberry and raspberry extracts). Additionally, it seemed that ZnO particles could be introduced into a HPMC coating carrier with a hydrophilic, active agent, e.g., chokeberry extract. However, zinc stearate can be introduced into the ethyl cellulose coating carrier containing a hydrophobic, active additive (raspberry extract) which is obtained through eco-friendly, supercritical CO_2_ extraction. As mentioned earlier, the synergistic effect of active agents can enable the application of a lower grammage of active coating to the surface of a PLA film resulting in an almost transparent, but active layer (Figure 1).

The aim of the study was to investigate the synergistic effect between selected plant extracts and zinc oxide, as well as selected plant extracts and zinc stearate. The purpose was to cover PLA films with active coatings based on HPMC containing the plant extract and ZnO with synergistic effect. An additional goal was to coat PLA with ethyl cellulose-based layers containing plant extract and zinc oxide and novel, EC based coatings containing RSE and zinc stearate. A determination of the antimicrobial properties of the covered films, as well as an investigation into the layer presence on PLA morphology (SEM, ATR-FTIR analysis) was also to be investigated.

## 2. Results and Discussion

### 2.1. Preliminary Research Work

**Figure 2:**
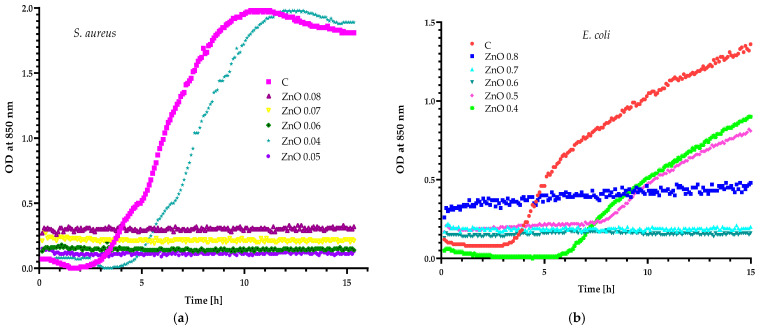
The impact of ZnO particles on the growth of: (**a**) *S. aureus* and (**b**) *E. coli*.

The preliminary experiments were carried out using two reference strains, Gram-positive *S. aureus* and Gram-negative *E. coli*. Previous investigations [32] demonstrated that, when 0.08 g of zinc oxide particles were added to 100 g of the 4% coating carrier, the resulting coating inhibited the growth of *S. aureus* and *E. coli* cells. Another study [24] determined that the addition of 0.04 g of ZnO led to the creation of a coating which inhibited the growth of the *S. aureus* strain and reduced the number of *E. coli* cells. However, the coating which contained a decreased amount of zinc oxide particles with the addition of geraniol showed higher activity than the layer that contained only particles, confirming a synergistic effect between ZnO particles and geraniol. Based on these observations, it was assumed that zinc oxide particles could demonstrate a synergistic effect, not only with a pure antimicrobial agent, such as geraniol, but also with the plant extract containing many active compounds. The tests that were performed showed that a fall in OD was not observed for *S. aureus* cultures, which were cultivated with ZnO particles (concentrations: 0.05–0.08%), confirming the growth inhibition. The 0.04% dispersion of zinc oxide particles in LB medium was observed to be inactive against Gram-positive bacterium (Figure 2a). The *E. coli* strain was seen to be less sensitive than *S. aureus* as not simply 0.04% but up to 0.05% dispersion of particles was not able to inhibit the growth of Gram-negative microorganisms (Figure 2b). The lack of activity in the 0.04% zinc oxide particles dispersion against both analysed strains led us to believe that this concentration of ZnO particles could be used to examine if there was either a synergistic or antagonistic effect between the particles and the selected plant extracts.

Research carried out previously by the authors [22] determined that the synergistic effect between plant extracts (such as *Scutellaria baicalensis* and *Glycyrrhiza* L.) as antimicrobial agents was noted. Moreover, other research [21] indicated that a synergism between *Uncaria tomentosa*, *Formitopsis betulina* extracts and zinc oxide particles was noted. Based on these conclusions, it was presumed that selected plant extracts (obtained using different methods) could offer a synergistic effect with zinc oxide particles.

Analysing the OD of microorganisms in a LB medium containing plant extracts over time, it was noted that there were differences between the initial values of the OD parameter, even if the number of bacterial cells (which were introduced into the test tubes containing LB with or without extracts) was the same (1.5 × 10^6^ CFU/mL). Interestingly, the initial OD values of microorganism cultures with black chokeberry extract was highest due to the dark brown colour of the extract. The lower, initial OD value was noted for cultures containing raspberry seed extract, due to its yellow hue. The lowest OD value was observed for microorganisms incubated in the LB medium without any extracts (control samples).

**Figure 3:**
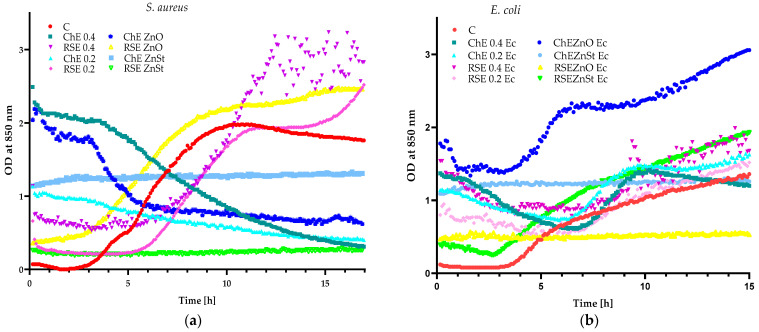
The impact of black chokeberry (ChE), CO_2_ raspberry seeds (RSE) extracts and extracts with the addition of ZnO and ZnSt on the growth of: (**a**) *S. aureus* and (**b**) *E. coli*.

The outcomes of preliminary investigations showed that the 4% and 2% *A. melanocarpa* extracts inhibited the growth of the *S. aureus* strain, because, as seen in Figure 3a, an OD increase for these cultures was not noted. However, both concentrations of black chokeberry extract were not effective against *E. coli* cells (an OD increase was observed) (Figure 3b). The CO_2_ extract of raspberry seed was seen as being inactive towards *S. aureus* and *E. coli* strains regardless of its concentration. Based on these results, a 2% chokeberry extract and 2% raspberry seed extract were selected for the next experiments. The 2% *A. melanocarpa* extract, as an agent which was inactive against *E. coli* cells, was able to demonstrate a synergistic effect with the addition of 0.04% of ZnO particles which were also found to be ineffective against Gram-negative bacteria. Alternatively, the 2% chokeberry extract which was active against *S. aureus* showed an antagonistic affect with ZnO particles. Additionally, 2% CO_2_ extract of raspberry seed was also selected for the next tests as ineffective against both microorganisms.

The *A. melanocarpa* extract was prepared using 70% ethanol. After ethanol evaporation, a water solution was obtained, meaning that the active compounds extracted from the plant were hydrophilic. It was thought that this extract might be introduced to the HPMC coating carrier which was water soluble [17,18]. However, to apply HPMC with *A. melanocarpa* extract on the surface of PLA, an emulsifier would have to be used due to biopolymer hydrophobicity. On the other hand, the CO_2_ extract of raspberry seed (a commercial product) was hydrophobic. This extract could be added to the EC which is a water insoluble [19] coating carrier. During the research it was assumed that the coating should have antimicrobial properties and also be transparent. This is why 2% chokeberry extract and 2% raspberry seed extract were selected for the next experiments. Mania et al. [28] reported that metal oxides, such as ZnO, can be an alternative to antibiotics that can lead to drug resistance. Additionally, they mentioned that zinc oxide, zinc gluconate, zinc chloride, zinc stearate and zinc sulfate are recognized as safe (GRAS). The authors added that zinc oxide is also less toxic to humans than silver particles. Based on these conclusions, and the previous study findings [24,32], zinc oxide particles were selected to see if there could be a synergistic effect between ZnO and *A. melanocarpa* and/or CO_2_ raspberry seed extracts. Additionally, zinc stearate (ZnSt) as a hydrophobic compound, insoluble in polar solvents was also selected to examine the potential synergistic effect between particles and the extracts. The 0.04% dispersion of ZnSt particles (in LB medium) was used to compare its impact on the activity of plant extracts in comparison to zinc oxide particles.

**Figure 4:**
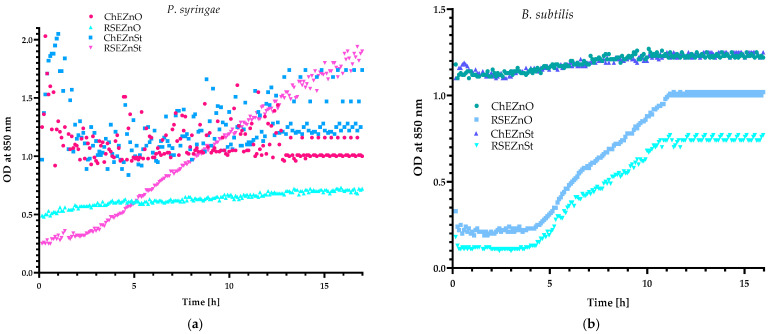
The impact of black chokeberry (ChE) and CO_2_ raspberry seed extracts (RSE) with the addition of ZnO and ZnSt on the growth of: (**a**) *P. syringae* and (**b**) *B. subtilis*.

Observations of the growth of selected Gram-positive and Gram-negative strains demonstrated that black chokeberry extract with the addition of ZnO was effective against *S. aureus*, *P. syringae* and *B. subtilis* strains, because neither an OD increase nor an OD decrease was noted (Figure 4a,b). Taking into account that the ChE was active against *S. aureus*, no antagonistic effect between ZnO and chokeberry extract was seen. Moreover, considering that the extract was not effective against *E. coli*, a synergistic effect between the particles and the ChE extract was also not observed (Figure 3a,b). Interestingly, chokeberry extract with ZnSt was seen to have antimicrobial properties against all of the analysed strains (Figure 3a,b and Figure 4a,b), confirming a synergistic effect between the particles and an *A. melanocarpa* extract.

An examination of the growth of selected microorganisms showed that the CO_2_ extract of raspberry seed with ZnO as an additive was only active against Gram-negative bacteria which indicated a synergistic effect between these agents. Furthermore, the RS extract with the addition of ZnSt was only active against *S. aureus*, showing a synergistic effect between the particles and the raspberry seed extract.

### 2.2. Antibacterial and Antiviral Properties Analysis of the Coatings

After the analysis of the synergistic effect between two selected extracts and particles in LB medium, an examination of the coatings containing extracts with the addition of zinc oxide and zinc stearate was performed. The coatings, based on a hydrophobic EC carrier containing water insoluble CO_2_ extract of raspberry seed was prepared with the addition of ZnO particles (AC1), and as a novel solution with ZnSt (AC2). The coating, based on a hydrophilic, HPMC carrier which contained black chokeberry extract, was prepared with the addition of zinc oxide particles (AC3) alone. It was not possible to prepare a coating dispersion with the zinc stearate due to its hydrophilic character.

**Figure 5:**
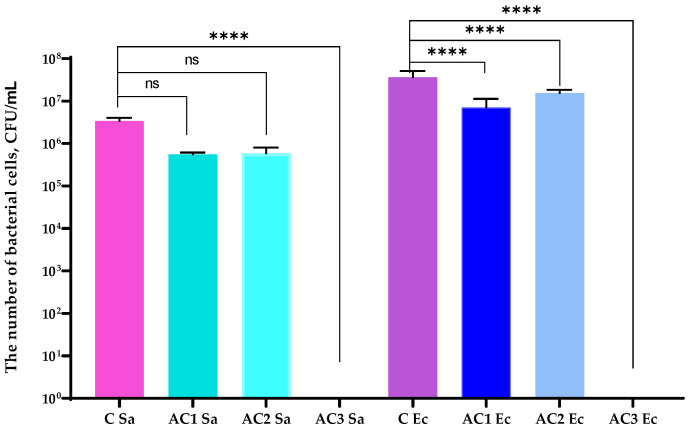
The growth of *S. aureus* (Sa) and *E. coli* (Ec) strains after its incubation with the active coatings. C—PLA film; AC1—PLA film coated with the EC carrier containing CO_2_ raspberry seed extract with addition of ZnO particles; AC2—PLA film coated with the EC carrier containing CO_2_ raspberry seed extract with addition of ZnSt particles; AC3—PLA film coated with the HPMC carrier containing black chokeberry extract with addition of ZnO particles. One-way ANOVA; ****—p < 0.0001, ns—*p* > 0.5.

The results of the research showed that both coatings based on ethyl cellulose containing raspberry seed extract decreased the number of *S. aureus* cells (≈1 log reduction) (Figure 5), even if the medium with the RS extract and ZnO was not effective against Gram-positive microorganisms. It should be mentioned that differences between the activity of AC1 and AC2 layers were not noted. The CO_2_ extracts from raspberry seed were confirmed as disrupting the plasma membrane or inhibiting the efflux pump [10,11,12]. The zinc oxide or zinc stearate could even have increased this mechanism of action due to a synergistic effect being observed. A previous study [10] demonstrated that an active coating containing CO_2_ raspberry seed extract was found to be more active against *S. aureus* than the AC1 and AC2 coatings from the current study; however, the HPMC, not an EC coating, was used. The reason could be the fact that the hydrophilic carrier (with emulsifier) released the hydrophobic extract rather than the EC coating. Better results were seen in the case of an active coating based on HPMC (AC3) which inhibited the growth of *S. aureus* completely. The LB medium containing the chokeberry extract with zinc oxide particles was also highly active. Comparing the effectiveness of AC1 and AC2 layers with the activity of AC3 coatings, it is possible that the PLA coated with the AC3 layer was the most active material. The anthocyanins extracted from *A. melanocarpa* were confirmed as increasing ROS generation, as well as the zinc oxide particles [2,6,7,8]. Both agents with a synergistic action effect might have induced oxidative stress in the microorganism cells and damaged the bacterial cell wall, which could have led to a leakage of intracellular material. Similar results were noted in the case of the *E. coli* and *P. syringae* strains. Previous research work [24] indicated that the HPMC coating containing geraniol or carvacrol with zinc oxide particles was the most active layer. Furthermore, a synergistic effect between the active compounds was observed. This study also confirmed that there was a synergistic effect between the active agents in the AC3 layer.

**Figure 6:**
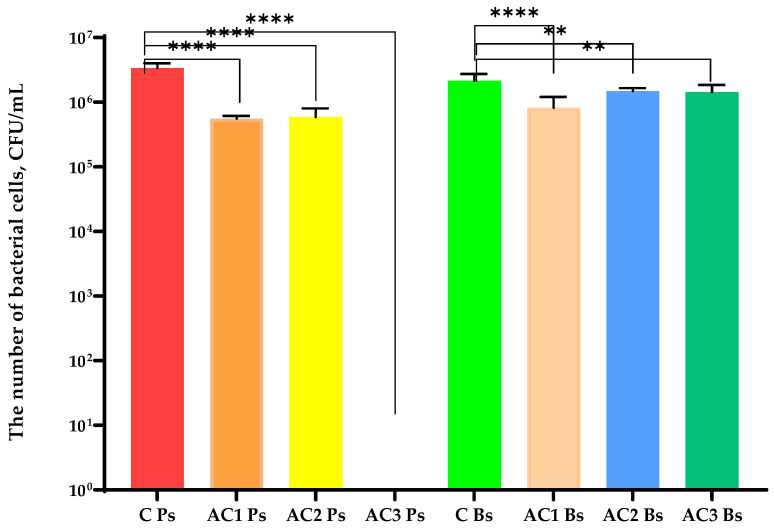
The growth of *P. syringae* (Ps) and *B. subtilis* (Bs) strains after its incubation with the active coatings. C—PLA film; AC1—PLA film coated with the EC carrier containing CO_2_ raspberry seed extract with addition of ZnO particles; AC2—PLA film coated with the EC carrier containing CO_2_ raspberry seed extract with addition of ZnSt particles; AC3—PLA film coated with the HPMC carrier containing black chokeberry extract with addition of ZnO particles. One-way ANOVA; **** p < 0.0001, ** p < 0.01.

The AC1 and AC2 coatings reduced the number of bacterial cells (Figure 6); however, the AC3 layer inhibited the growth of Gram-negative bacteria. The hydrophilic character of this coating carrier which led to a much faster release of active compounds could have been the reason. The findings of a previous study [10] revealed that an HPMC coating containing an RS extract was even less effective against Gram-negative bacteria than AC1 and AC2 coatings. The outcomes also indicated that none of the described coatings inhibited the growth of the *B. subtilis* strain (Figure 5). Moreover, the AC1 coating was seen as the most effective layer against Gram-positive bacilli cells. However, the decrease in the cell number was less than 1 log. Furthermore, the HPMC [10] coating containing the RS extract was found to be more effective against *B. subtilis* strains than the AC1 and AC2 layers.

**Figure 7:**
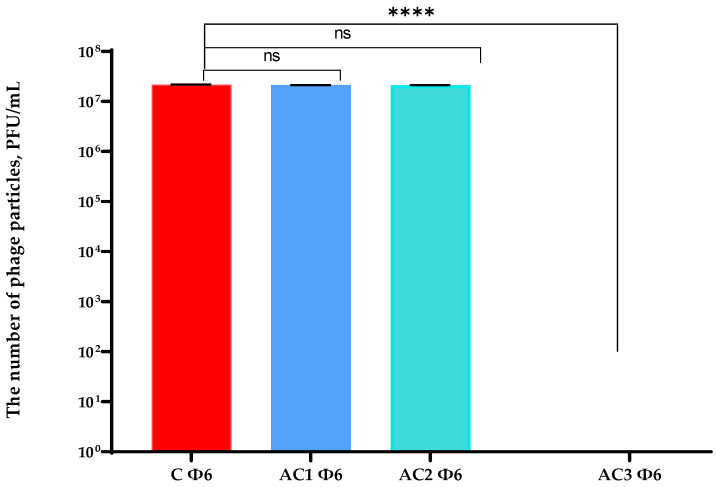
The titre of phi6 phage after its incubation with the active coatings. C—PLA film; AC1—PLA film coated with the EC carrier containing CO_2_ raspberry seed extract with addition of ZnO particles; AC2—PLA film coated with the EC carrier containing CO_2_ raspberry seed extract with addition of ZnSt particles; AC3—PLA film coated with the HPMC carrier containing black chokeberry extract with addition of ZnO particles. One-way ANOVA; **** p < 0.0001, ns—*p* > 0.5.

**Figure 8:**
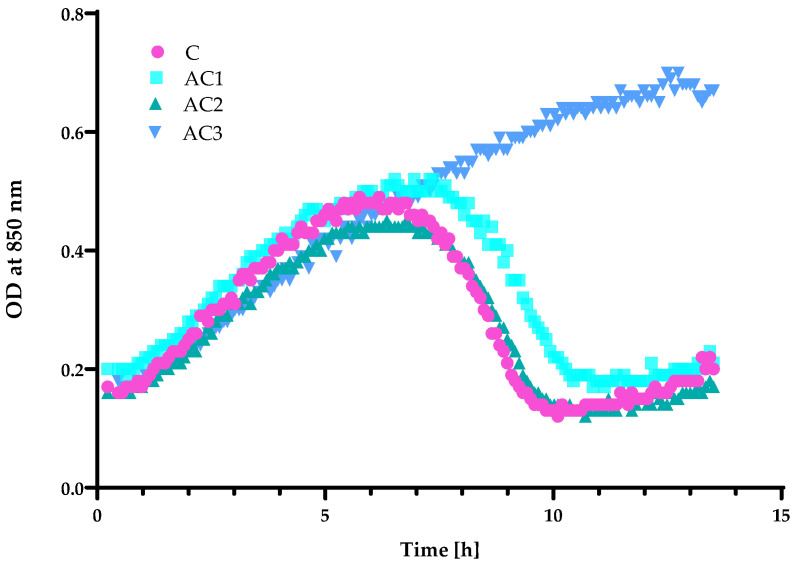
The OD over time *P. syringae* with the phi6 bacteriophage after its incubation with the active coatings. C—PLA film; AC1—PLA film coated with the EC carrier containing CO_2_ raspberry seed extract with addition of ZnO particles; AC2—PLA film coated with the EC carrier containing CO_2_ raspberry seed extract with addition of ZnSt particles; AC3—PLA film coated with the HPMC carrier containing black chokeberry extract with addition of ZnO particles.

An analysis of the bacteriophage titre (Figure 7) demonstrated that AC1 and AC2 coatings were not active against bacterial viruses, even if the phage was enveloped by a lipid layer and both of the coatings had a hydrophobic character. Additionally, a fall in OD was observed after 7 h of the cultivation of the host (*P. syringae*) with the Φ6 particles that were incubated with the PLA film (uncovered, control sample) and/or PLA films coated with the AC1 and AC2 layers (Figure 8). These outcomes confirmed that the AC1 and AC2 coatings were not active against Φ6 particles. A previous study confirmed [10] that the HPMC coating with the addition of the RS extract deactivated phi6 particles, confirming that it had antiviral properties. In addition, the HPMC coating containing zinc oxide particles and geraniol or carvacrol [24] demonstrated moderate antiviral activity; however, the synergistic effect between active agents was confirmed.

Contrary results were noted in the case of the active coating based on HPMC which contained chokeberry extract and zinc oxide particles (Figure 7 and Figure 8). An OD fall was not noted and host growth was observed, meaning that bacteriophage particles were inactivated/eliminated by the AC3 coating (Figure 8).

### 2.3. Microscopic Analysis of Films Covered with the Active Coatings

Scanning electron analysis of the zinc oxide and zinc stearate powders showed particles of various shapes and diameters. Additionally, there was a tendency for the particles to form agglomerates (Figures S1 and S2). Tania I.S. and Ali M. [33] demonstrated that the sizes of particles of zinc oxide (directly after centrifugation, washing and drying) were in the range of 49–103 nm. Based on previous work [24,27], it was assumed that to avoid the formation of agglomerates in the coating carriers, the zinc oxide and zinc stearate were sonicated.

**Figure 9:**
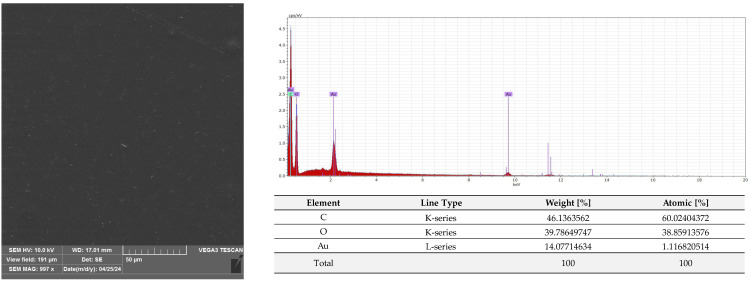
Morphology and EDS spectrum of PLA film.

**Figure 10:**
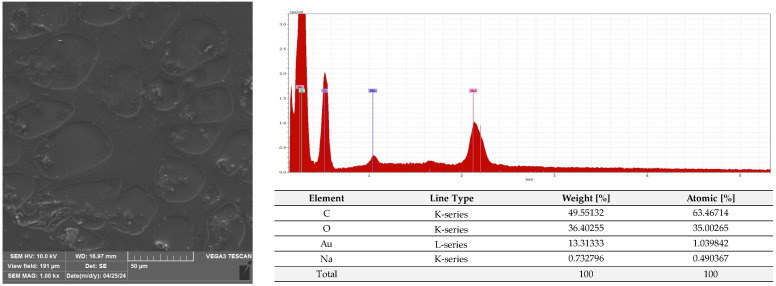
Morphology and EDS spectrum of PLA covered with HPMC coating carrier with emulsifier.

**Figure 11:**
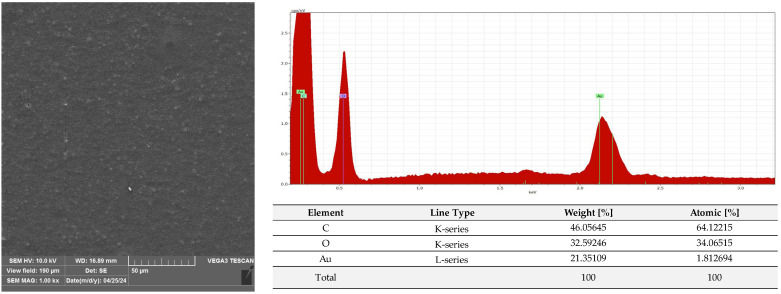
Morphology and EDS spectrum of PLA covered with EC coating carrier.

**Figure 12:**
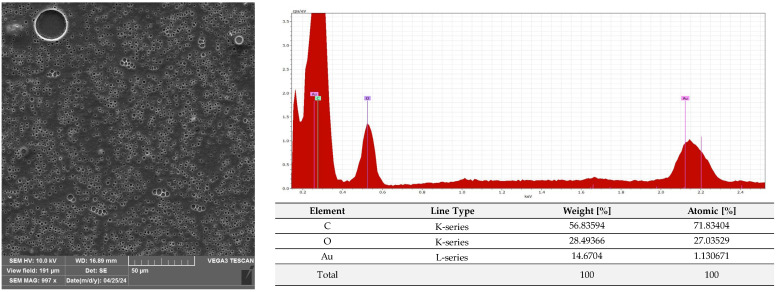
Morphology and EDS spectrum of PLA covered with AC1 coating.

**Figure 13:**
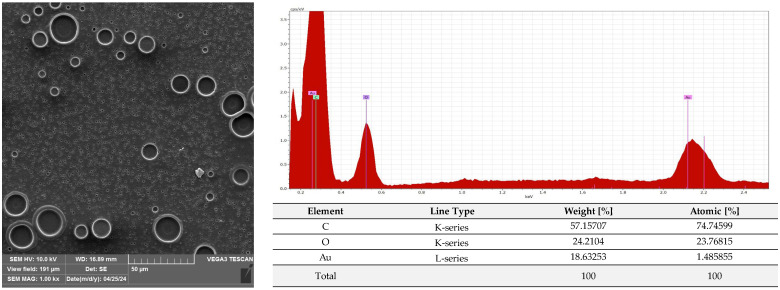
Morphology and EDS spectrum of PLA covered with AC2 coating.

A microscopic analysis was carried out to capture a view of the surface of the PLA film and the AC1, AC2 and AC3 coatings applied to the PLA surface. Figure 9 and Figure S3 show that the PLA film exhibited a slightly homogeneous, smooth surface. Moreover, an EDS analysis confirmed that uncoated PLA film showed the elemental characteristic peaks of the PLA molecule, i.e., carbon (C) and oxygen (O). Strong Au signal peaks came from the gold coating prior to SEM investigation. Similar results were noted by Jamnongkan, T. et al. [34]. Analysing the coatings which were applied to the PLA surface, it was shown that HPMC (due to its hydrophilic character) did not cover (homogenously) the PLA surface despite the addition of an emulsifier (Figure 10 and Figure S4), while ethyl cellulose as a hydrophobic carrier offered better adhesion to the PLA surface. As clearly seen in Figure 11 and Figure S5, PLA was thoroughly and homogenously covered with the EC layer. Furthermore, an EDS examination demonstrated the differences between the elemental compositions of uncoated PLA and film covered with the HPMC and EC layers, which confirmed the presence of coatings on the surface of PLA (coating surfaces consisted of a lower amount of oxygen and a greater quantity of carbon than compared to neat PLA). Additionally, as emphasized in Figure 10, sodium was detected on the surface of the HPMC. The addition of raspberry seed extract and zinc oxide or zinc stearate had an influence on the surface of the coatings. There were holes/pores visible in the AC1 (Figure 12 and Figure S6) and AC2 layers (Figure 13 and Figure S7) which could lead to the release (perhaps fast release) of active compounds, and as a result could lead to the lower activity of the AC1 and AC2 coatings. An Energy Dispersive Spectroscopy analysis showed elemental peaks in the AC1 and AC2 coatings, i.e., C and O (Figure 12 and Figure 13). Strong signal peaks of Au came from the gold coating prior to SEM examination. The zinc oxide and zinc stearate were not detected by EDS which could be confirmation of their release from the coating. Similar SEM micrographs of the coatings based on ethyl cellulose were observed by Baghi et al. [35]. The authors explained that the presence of heterogeneous pores could be due to the rapid evaporation rate of the ethanol during the drying process. Kaloper et al. [36] covered polymer film with a chitosan coating with the addition of blackberry extract. The authors observed that cracks and large agglomerates were present on the surface of coating. The authors suggested that colloids from the extracts, such as multimolecular colloids, polyphenol macromolecules or inorganic elements, such as plant minerals and polyphenols, were responsible for agglomerate formation. The HPMC coating containing RS extract (without zinc oxide particles or zinc stearate), which was described in a previous study [10], was more microbiologically active than AC1 and AC2 coatings (a coating from the current research, based on EC). This can be attributed to the faster release of the active compounds by the HPMC layer (in comparison to EC coating). A SEM analysis indicated that the surface of the active HPMC coating was not homogeneous. Moreover, the spherical molecules of the hydrophobic RS extract were clearly visible in the coating carrier [10].

**Figure 14:**
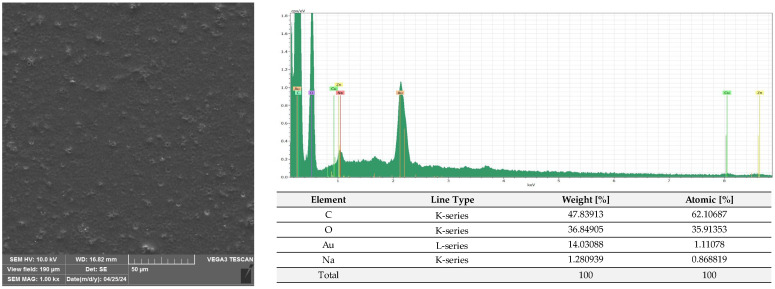
Morphology and EDS spectrum of PLA covered with AC3 coating.

Comparing the HPMC coating with the AC3 layer, it was determined that the addition of chokeberry extract and zinc oxide particles improved the adhesion of the coating to the PLA surface. SEM analysis made it possible to observe the distribution between the active agents in the coating. As seen in Figure 14 and Figure S8, the AC3 layer was thoroughly, homogenously and uniformly applied to the surface of the PLA film. In addition, it was the most effective layer (microbiologically) that inhibited the growth of the *S. aureus*, *E. coli* and *P. syringae* strains. The HPMC with the *Scutellaria baicalensis* and *Glycyrrhiza L*. extracts (with emulsifier) was applied to the surface of the polyethylene film [37]. Microscopic examination showed that the PE surface was thoroughly, uniformly and homogenously covered with the active coating. This observation was similar to the current tests. The EDS test demonstrated the elemental characteristic peaks of the AC3 coating, i.e., carbon, oxygen and a low amount of Na (Figure 14). The spectrum also showed the presence of the element Zn. However, due to a trace amount of zinc oxide in the AC3 coating (lower that 1%), zinc was detected, but a quantified analysis was not possible. Jamnongkan, T. et al. [34] detected Ag on the fabricated electrospun PLA nanofibers. The authors also performed quantified analysis of the elements using the EDS method, though the addition of the particles to the PLA fibres was higher than 1%. Similarly, Li, Q.S., et.al. [38], detected and quantified Zn on polypropylene nanofibers, though the addition of zinc oxide nanoparticles was also higher than 1%.

**Figure 15:**
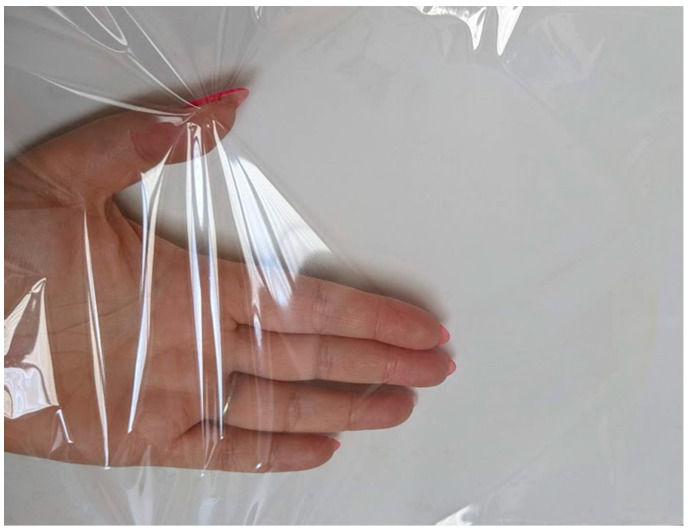
The surface of AC3 coatings.

To summarize, there is an increasing need to develop natural and effective antimicrobial coatings which are required to be transparent. The use of dark brown plant extract led to a decrease in coating transparency. The solution may be to create a coating with a much lower amount of extract or a thinner coating. However, this may lead to a decrease in their antimicrobial activity. A previous study [24] demonstrated that there was a synergistic effect between geraniol or carvacrol and zinc oxide particles. Zheng et al. [39] also confirmed a synergy between these two active agents. Mania et al. [28] suggested that zinc oxide as an additive may facilitate the distribution of active compounds in a polymer or biopolymer and lead to a synergistic effect between active compounds. This research has confirmed these suppositions. These results led to the creation of a thin, transparent (Figure 15) AC3 coating which was homogenously applied to the PLA surface and, additionally, the AC3 layer was also found to have antibacterial and antiviral properties. Carvalho et.al. [40] reported that the thinner coatings containing zinc oxide particles had a more compact morphology in comparison to the thickest. In addition, the authors noted that an increase in coating thickness induced the formation of less compact layers. However, to obtain highly effective, thin antimicrobial coatings, Ag nanoparticles had to be added to the coatings with zinc oxide nanoparticles (confirming the synergistic effect between active agents). Sarhadi et al. [41] investigated the antimicrobial properties of active materials based on starch and nanoZnO containing various concentrations of *Ferula gummosa* essential oil. The authors reported that the addition of ZnO nanoparticles improved the thickness of the aforementioned materials. Moreover, the addition of zinc oxide nanoparticles to the starch matrix resulted in antimicrobial effectiveness against *S. aureus* and *E. coli*. Interestingly, the highest antimicrobial properties were observed in starch containing both active agent nanoparticles and *F. gummosa*. The synergistic effects of the antimicrobial materials are presented in Table 1.

### 2.4. Results of ATR-FTIR Analysis

**Figure 16:**
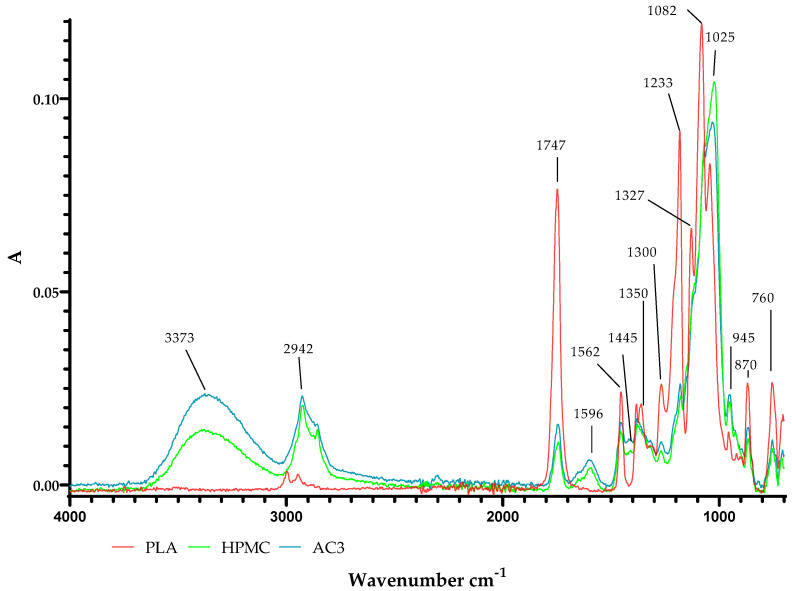
ATR-FTIR spectra of the PLA film, PLA film coated with the HPMC coating and PLA film covered with the active coating containing chokeberry extract and ZnO (AC3).

The PLA film and PLA film coated with the AC1, AC2 and AC3 coatings were evaluated via FTIR. Figure 16 shows the FTIR spectrum for the active coating and pure carrier (HPMC), as well as the PLA sample. Comparing the pure coating carrier (HPMC) and the layer containing antimicrobial agents with the PLA film, it was observed that a broad band around the range of 3900–3020 cm^−1^ assigned to axial stretching OH groups was noted with a peak maximum at 3373 cm^−1^ for both the HPMC and AC3 layers (with higher intensity for the coating modified with chokeberry extract and zinc oxide particles). In the case of the 2942 cm^−1^ peak, peak consistency with absorption was observed, stimulated by C–H stretching single bonds. Alternatively, a spectra peak at 1747 cm^−1^ was seen, stimulated by C=C stretching, double bonds. Nascimento Da Silva et al. [42] suggested that the peak at 2942 cm^−1^ corresponds to stretching C–H bonds of HPMC chains, while the peak at 1747 cm^−1^ corresponds to bending C=O of the carboxylic acid and oleic acid. Moreover, the presence of the coatings on the surface of PLA may be confirmed with small peaks at 1596 cm^−1^ and 1445 cm^−1^ for HPMC and AC3 (also with higher intensity for the coating modified with chokeberry extract and zinc oxide particles), respectively, which were not noted in the uncoated PLA. The peak at 1445 cm^−1^ could be attributed to the C=C stretching vibration of the aromatic ring. This peak, according to Halasz et al. [43] and Ćujić et al. [44] could be related to the *A. melanocarpa* extract. Janković et al. [45] mentioned that the most prominent bands (≈1600–1700 cm^−1^) seen in chokeberry extracts were amide I, detected in the chokeberry extract. The peak at 1596 cm^−1^ which was noted in this work was almost in this range, suggesting that it could be related to the *A. melanocarpa* extract. Kalpana et al. [46] reported that to recognize the functional groups of ZnO particles, absorption peaks at 3199.91, 1587.42, 1315.46, 1074.35, 1041.56 and 823.60 cm^−1^ should be detected. These peaks were not observed in the current study. However, the amount of zinc oxide particles which was introduced into the coating carrier to create the active coating was trace.

**Figure 17:**
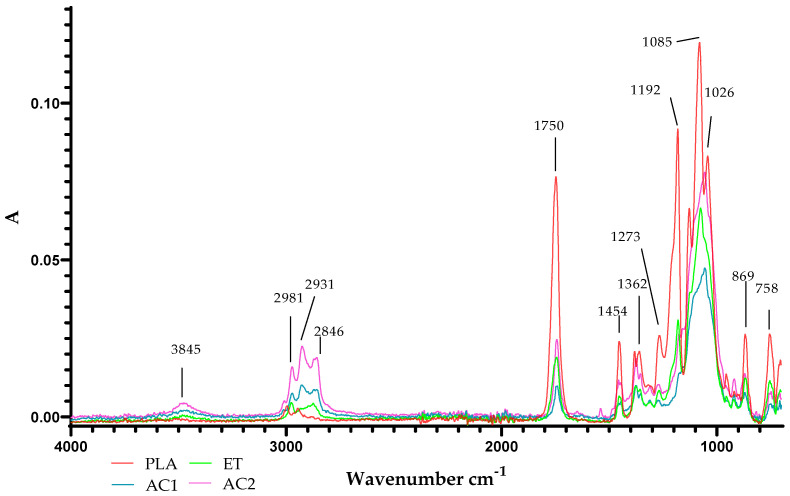
ATR-FTIR spectra of the PLA film, PLA film coated with the EC coating and PLA film covered with the active coating containing CO_2_ raspberry seed extract and particles of ZnO (AC1) and ZnSt (AC2).

When analysing ethyl cellulose as a coating carrier and the two layers (AC1 and AC2) containing antimicrobial agents, as well as the PLA film, it was noted that the broad band in the range of 3900–2800 cm^−1^ assigned to axial stretching OH groups was observed with a peak maximum at 3845 cm^−1^ for EC, and both AC1 and AC2 layers (Figure 17). Sachadyn-Król et al. suggested that this could correspond to phenolic compounds [47] from a raspberry extract. The peaks at 2981, 2931 and 2846 cm^−1^ attributed to –CH and the following peaks at 1750, 1454, and 1362 cm^−1^ corresponded to C= O, –CH_2_, and –CH_3_, respectively. The broad distinct peak at 1085 cm^−1^ due to the C–O–C stretch corresponded to cyclic ether. Moreover, the peak at 869 cm^−1^ referred to –CH_2_ and –CH, respectively, and indicated the presence of epoxy groups, confirmed by Baghi et al. [35]. It is worth mentioning that the peaks at 2846 cm^−1^ were only present in the case of the EC, AC1 and AC2 layers which confirmed that the coatings were applied to the surface of the PLA.

## 3. Materials and Methods

### 3.1. Materials

The microorganisms used to investigate antibacterial and antiviral activity analysis were purchased from the Leibniz Institute Deutsche Sammlung von Mikroorganismen und Zellkulturen (DSMZ, Braunschweig, Germany) collection. The bacteria used in these experiments were Gram-positive bacterial strains such as: *B. subtilis* DSMZ 1090, *S. aureus* DSMZ 346, and Gram-negative bacterial strains: *E. coli* DSMZ 498 and *P. syringae* van Hall 1902 DSM 21482. The *P. syringae* strain was used as a bacterial virus’s (Φ6) host. Bacteriophage phi 6 DSM-21518 was used as a SARS-CoV2 surrogate to determine antiviral activity of the obtained, active coatings/coverings.

The ethanol (98%, Warchem, Trakt Brzeski, Poland) was used to obtain *A. melanocarpa* extracts. PLA film (20 μm) (CBIMO, Szczecin, Poland) was used as material to cover it with the active coatings. The hydroxypropyl methyl cellulose-HPMC (Dow Wolff Cellulosics GmbH, Bomlitz, Germany) and ethyl cellulose (POL-AURA^®^, Szczecin, Poland) were used as coating carriers in the research work. Caprylyl/Capryl glucoside and Decyl Glucoside (Greenaction Sp. z o.o., Kielce, Poland) were used as emulsifiers. *A. melanocarpa* (Kawon, Gostyń, Poland) was used to prepare ethanolic plant extract. Ecological CO_2_ extract of raspberry seeds (ECOSPA, Warszawa, Poland) was used as active agent to prepare active coatings. Zinc oxide particles and zinc stearate (Permedia, Lublin, Poland) were used to increase antimicrobial effectiveness of the plant extracts. To investigate the antimicrobial effectiveness/properties of the extracts and of the active layers, MacConkey agar, TSA, Luria-Bertani (LB) and TSB broths (Merck, Darmstadt, Germany) were prepared. The preparation of all mediums was carried out according to the manufacturer’s protocols (all mediums were weighed according to Merck instructions, introduced into 1 L of distilled water and sterilized 15 min at 121 °C).

### 3.2. Methods

#### 3.2.1. Extracts Preparation

Dry *A. melanocarpa* fruits were used to obtain the ethanolic plant extract. As first step of the experiment, 50 g of the dry fruits of *A. melanocarpa* was introduced into a TM6 Thermomix (VORWERK, Wrocław, Poland). The fruits were ground to a powder (7600 rpm, 30 s). As the next step, the fruits were put into 100 mL of 70% ethyl alcohol. Then, the bottle containing ethanol solution with black chokeberry fruits was put into a microwave (Amica, Wronki, Poland) for 10 min at output power of 81%. The bottle was then transferred to a shaker (Ika, Staufen im Breisgau, Germany) to continue the extraction for 1 h at 70 °C (150 rpm). The slightly modified extraction was carried out according to methods described by Kubra et al. and Mandal et al. [48,49]. When the extraction was completed, the fruits were separated from the extracts using a Büchner funnel. Finally, chokeberry fruits were filtered through a 0.2 μm filter and evaporated (to remove ethanol) to obtain a water *A. melanocarpa* solution with a dry mass = 59.75% and pH = 4.13. The pH of the commercial CO_2_ raspberry seed extract was 4.32.

#### 3.2.2. Preliminary Analysis of Extracts and Zinc Oxide

As a first step of the preliminary tests, the effectiveness of zinc oxide particles was investigated. Next, the antibacterial activity of *A. melanocarpa* extract and CO_2_ extract of raspberry seed was measured. It was assumed that a preliminary, antibacterial property analysis would only be performed against *S. aureus* and *E. coli* strains to see the minimal inhibition concentration (MIC) of the active agents in real time. These experiments were performed to determine the concentrations of the extracts and zinc oxide particles which would be used in the next tests. A current antibacterial activity was analysed as described in a previous manuscript [23].

The *S. aureus* strain was pre-grown on TSA; however, *E. coli* cells was pre-grown on MacConkey agar (for 24 h, at 37 °C). Then, the biomass of both strains (separately) was suspended in a sterile 0.85% NaCl solution to obtain bacterial cells inoculum of 1.5 × 10^8^ CFU/mL. As a next step, a preparation of LB medium containing ZnO particles (concentrations: (wt: 0.08%, 0.07%, 0.06%, 0.05% and 0.04%) was carried out, while simultaneously, 4% and 2% (wt) solutions of *A. melanocarpa* extract and 4% and 2% (wt) dispersions of CO_2_ extract of raspberry seed were prepared (separately) in 10 mL of LB medium in test tubes. The samples were mixed with a vortex stirrer (150 rpm, Ika, Legnica, Poland) for 1 min. The suspended biomass of *S. aureus* or *E. coli* (100 μL) was added to sterile test tubes which contained LB with extracts or ZnO and was further mixed in a vortex (Ika, Legnica, Poland) for 1 min. The LB medium with *S. aureus* or *E. coli* which did not contain any extracts or ZnO was analysed as control samples. Then, antibacterial effectiveness (the analysis of bacterial growth rate in real time) of all solutions and dispersions was investigated. The test tubes containing LB broth with the extracts or particles of zinc oxide or LB medium without active agents with the addition of *S. aureus* or *E. coli* were introduced into BioSan bioreactors (BS-010160-A04, BioSan, Riga, Latvia) and incubated at 37 °C.

#### 3.2.3. Analysis of Synergistic Effect between Extracts and Zinc Oxide and Stearate

The 2% *A. melanocarpa* extract solution, 2% CO_2_ extract of raspberry seed dispersion and 0.04% zinc oxide particles dispersion in LB medium (due to their lack of activity or low activity against *S. aureus* or *E. coli*) were selected to investigate the antimicrobial properties of the extract mixtures and ZnO particles, as well as to determine the synergistic effect between these active agents. The antibacterial effectiveness of the active agents on bacterial cells was analysed, as well as the growth of bacteria in real time in the presence of active compounds.

The inoculum of 1.5 × 10^8^ CFU/mL of *S. aureus* strain was prepared as described above. A 4% (wt) solution of *A. melanocarpa* extract and a 4% CO_2_ extract of raspberry seed dispersion in LB medium in test tubes were prepared. Then, the samples were mixed (150 rpm, Ika, Legnica, Poland) for 1 min. Later, a 0.08% (wt) dispersion of ZnO particles was prepared. It was assumed that not only the zinc oxide particles, but also the zinc stearate could have an influence on the antimicrobial activity of *A. melanocarpa* and raspberry seed extracts (resulting in a synergistic effect). As a consequence, 0.08% zinc stearate in LB medium was prepared. Then, the LB medium containing fruit extracts was mixed with the zinc oxide and zinc stearate dispersions (separately) at a 1:1 ratio (to obtain a 2% final concentration of extracts with the addition of 0.04% zinc oxide or 0.04% zinc stearate dispersions). All of the aforementioned dispersions were mixed in a vortex (Ika, Legnica, Poland) for 1 min. The suspended biomass of *S. aureus* or *E. coli* (100 μL) was added to sterile test tubes, which contained LB with extracts at concentrations of 2% and 4%, and was further mixed in a vortex (Ika, Legnica, Poland) for 1 min. Subsequently, the *S. aureus*, *B. subtilis*, and *P. syringae* strains were pre-grown on TSA (for 24 h, at 37 °C and 28 °C); however, *E. coli* cells were pre-grown on MacConkey agar (for 24 h, at 37 °C). Next, the biomass of all strains was suspended separately in a sterile 0.85% NaCl solution to obtain a bacterial cell inoculum of 1.5 × 10^8^ CFU/mL. The suspended biomass of the aforementioned bacterial strains (100 μL) was added to test tubes with active dispersions. The test tubes were then introduced into BioSan bioreactors (BS-010160-A04, BioSan, Riga, Latvia) and incubated for 24 h at 37 °C (*S. aureus*, *B. subtilis*, *E. coli* strains) or 28 °C (*P. syringae*).

#### 3.2.4. Coating System Preparation

The 8% HPMC solution in distilled water (92 g) was prepared, which would act as a coating carrier. The carrier was mixed for 1 h using a magnetic stirrer (Ika, Warsaw, Poland) at 1500 rpm. When the HPMC solution was homogenous, 2 g of caprylyl/capryl glucoside and 2 g of decyl glucoside were added into the HPMC. The carrier was mixed for 10 min. Then, 96 g of HPMC with emulsifiers was mixed with 4 g of *A. melanocarpa* extract solution and stirred (15 min, 1500 rpm, RT) (Ika, Warsaw, Poland). A 4 wt% *A. melanocarpa* extract solution in HPMC was then obtained. As a next step, 0.08 g of ZnO particles was introduced into 99.92 g of distilled water and mixed for 30 min (750 rpm, RT). After stirring, the dispersion was then sonicated for 30 min (sonication parameters: power: 400 W, frequency: 24 kHz, amplitude: 20%, cycle: 0.5). After sonification, the carrier containing the chokeberry extract was mixed with ZnO particles (wt, 1:1). Then, the system with active agents was mixed for 1 h (Ika, Warsaw, Poland) at 1500 rpm.

As a next step, two beakers of 8% ethyl–cellulose (EC) solution in ethanol (96 g), as coating carriers, were prepared. The coating carriers were mixed for 1 h using a magnetic stirrer (Ika, Warsaw, Poland) at 1500 rpm to obtain homogenous solutions. Then, 96 g of EC was mixed with 4 g of CO_2_ extract of raspberry seed. The dispersions were stirred (15 min, 1500 rpm, RT) (Ika, Warsaw, Poland). Two 4 wt% raspberry extract solutions in EC were then obtained. As a next step, 0.08 g of ZnO particles was introduced into 99.92 g of ethanol and mixed for 30 min (750 rpm, RT). After stirring, the dispersion was then sonicated for 30 min (sonication parameters: amplitude: 20%, cycle: 0.5). At the same time, 0.08 g of zinc stearate was introduced into 99.92 g of ethanol and mixed for 30 min (750 rpm, RT). After stirring, the dispersion was sonicated as described above. After sonication, the carriers containing raspberry extract were mixed with ZnO particles and zinc stearate separately. Then, the systems with the active agents were mixed for 1 h (Ika, Warsaw, Poland) at 1500 rpm. In addition, 4% HMPC (in distilled water) and 4% EC (in ethanol) carriers were prepared.

The PLA was covered with the active coatings using Unicoater 409 (Erichsen, Hemer, Germany) at room temperature with a 25 μm diameter roller. The active layers applied to the PLA were then dried for 10 min at a temp. of 40 °C. The following grammages: 1.74 g/m^2^ (AC1, layer based on EC containing 2% of CO_2_ extract of raspberry seed and 0.04% of ZnO particles); 1.78 g/m^2^ (AC2, layer based on EC containing 2% of CO_2_ extract of raspberry seed and 0.04% of zinc stearate); and 1.76 g/m^2^ (AC3, layer based on HMPC containing 2% of chokeberry extract and 0.04% of ZnO particles), respectively, were applied to the PLA film. Unmodified PLA films were analysed as control samples (C). Additionally, coating carriers (HMPC and EC) were applied to the PLA films. The PLA films covered with HMPC (C1) and EC (C2) non-active layers were also analysed as control samples. PLA was coated with the active compositions on one side of the biopolymer material. Nevertheless, the coverings containing active agents could have been applied to both sides of films: an internal layer/covering offering antibacterial properties and an external layer/covering with antiviral properties. The active (AC1, AC2, AC3) and non-active (control) foil samples (C1, C2, C3) were cut into squares (3 cm × 3 cm).

#### 3.2.5. Antibacterial and Antiviral Analysis of the Coatings

The antibacterial effectiveness/activity of PLA films covered with AC1, AC2, AC3 active layers, C2 and C3 non-active layers and non-covered PLA foil samples was determined according to the ASTM E 2180-01 standard [50]. To investigate the antiviral effectiveness/activity of the active and non-active layers described above, the Φ6 bacteriophage lysate was prepared as Bhetwal et al. [51] and Bonilla et al. [52] reported. The antiviral effectiveness of AC1, AC2, AC3 layers compared to the non-covered PLA films (C1) and non-active C2 and C3 coatings were investigated according to a modified ISO 22196-2011 standard [53]. As a next step, Φ6 lysate particles amplification was carried out as Skaradzińska et al. described [54]. To examine the host (*P. syringae*) rate in real time, after its incubation/contact with the active and non-active coatings applied to the surface of the PLA films, Φ6 particles were left in contact (cultivated) with the active AC1, AC2 and AC3 layers (each coating separately) and control samples (C1, C2, C3) according to the ISO 22196-2011 [53] standard using BioSan bioreactors (BS-010160-A04, BioSan, Riga, Latvia). The host/*P. syringae* overnight inoculum/culture was added to 10 mL of LB broth and cultivated at 28° until OD = 0.2 (optical density). Six phi6 lysates were amplified in the bacterial culture (1 lysate sample—after its cultivation with the AC1 coating; 1 lysate sample—after cultivation with the PLA covered with AC2 coating; 1 lysate—after its cultivation with the sample coated with the AC3 active layer; 1 lysate—after its cultivation with the PLA coated with the C2 non-active covering; 1 lysate—after its cultivation with the film coated with the C3 non-active coating; and, finally; 1 lysate—after its cultivation with the C1, non-coated sample). As a next step, 10 µL of bacteriophage was introduced to a *P. syringae* culture (when OD was 0.2) and cultivated for 24 h at 28°. Six experiments were carried out simultaneously.

The statistical exploration of PLA films and the films covered with C2. C3, AC1, AC2 and AC3 layers was examined using analysis of variance (one-way ANOVA). Statistical significance was noted when *p* < 0.05. Significance was determined by using GraphPad Prism 8 (GraphPad Software, San Diego, CA, USA).

#### 3.2.6. SEM and EDS Analysis

The PLA film (C1) and the film coated with non-active C2, C3 and AC1, AC2 and AC3 active layers were examined using a scanning electron microscope (SEM). All samples were placed on pin stubs and covered with a layer of gold in a sputter coater at 24 °C (Quorum Technologies Q150R S, Laughton, East Sussex, UK). SEM micrographs were taken using a Vega 3 LMU microscope (Tescan, Brno-Kohoutovice, Czech Republic). The goal of this examination was to demonstrate that the PLA films had been thoroughly coated with the AC1, AC2 and AC3 active layers. The SEM analysis was carried out through the use of a tungsten filament with an accelerating voltage of 10 kV. Energy Dispersive Spectroscopy (EDS) was used for the elemental analysis of the neat and coated PLA under an electron Vega 3 LMU microscope with Bruker’s analyzer/detector (BRUKER Polska, Poznań, Poland) with an accelerating voltage of 20 kV.

#### 3.2.7. ATR-FTIR Analysis

Fourier-transform infrared (FTIR) spectrum of the unmodified and covered PLA film (both sides of the films) was examined using FT-IR spectrophotometer (Perkin Elmer Spectrophotometer (Waltham, MA, USA), Spectrum 100), operated at a resolution of 4 cm^−1^ and with 16 scans. The spectrum was recorded at a wavenumber of 4000–600 cm^−1^.

## 4. Conclusions

The performed experiments demonstrated that black chokeberry extract (ChE) and zinc oxide particles were effective against *S. aureus*, *P. syringae* and *B. subtilis* strains. Moreover, the ChE with zinc stearate (ZnSt) was active against all analysed strains. The HPMC with ChE and ZnO as additives had antimicrobial properties against *S. aureus*, *P. syringae* and *E. coli* strains. The ChE was confirmed to inhibit the growth of all of the examined bacterial strains. When analysing the coatings based on EC with CO_2_ extract of raspberry seed (RSE) and ZnO, it was noticed that they were only effective against Gram-negative bacteria. The results of the research work confirmed that AC1 (EC with RSE with ZnO) and AC2 (EC with RSE with ZnSt) coatings were not active against phi6 bacteriophage. The HPMC coating containing an AC3 layer (ChE and ZnO) eliminated Φ6 particles, confirming its antiviral effectiveness. Additionally, the presence of the active (AC1, AC2 and AC3) coatings was confirmed by SEM and FTIR analysis.

## Figures and Tables

**Table 1:** The active materials containing antimicrobial agents with synergistic effect.

Material	Synergistic Effect	Active Agent		Ref.
		1	2	
Polyethylene film (PE)	+	zinc oxide	geraniol/carvacrol	[24]
Modified PLA composites	+	nanocrystal–zinc oxide	carvacrol	[28]
LDPE/LLDPE	+	zinc oxide nanoparticles	zinc stearate nanoparticles	[36]
PET (Polyethylene terephthalate)	+	zinc oxide nanoparticles	Ag nanoparticles	[37]
Starch nanocomposite	+	zinc oxide nanoparticles	*Ferula gummosa* essential oil	[38]

## Data Availability

Data are contained within the article and Supplementary Materials.

## Supplementary Materials

The following supporting information can be downloaded at: https://www.mdpi.com/article/10.3390/molecules29153493/s1, Figure S1–S8: The Influence of Zinc Oxide and Zinc Stearate on the Antimicrobial Activity of Coatings Containing Raspberry and Chokeberry Extracts”.
