# Supplementary material for: The Influence of Zinc Oxide and Zinc Stearate on the Antimicrobial Activity of Coatings Containing Raspberry and Chokeberry Extracts
Source: Molecules. 2024 Jul 25;29(15):3493. doi: 10.3390/molecules29153493 (PMC11314005; doi:10.3390/molecules29153493)
Supplement: Supplementary file 1 [file molecules-29-03493-s001.zip › molecules-3054426-Supplementary Material.pdf]

SUPPLEMENTARY INFORMATION

The Influence of Zinc Oxide and Zinc Stearate on the Antimicrobial Activity of Coatings Containing Raspberry and Chokeberry Extracts

Małgorzata Mizielińska and Artur Bartkowiak

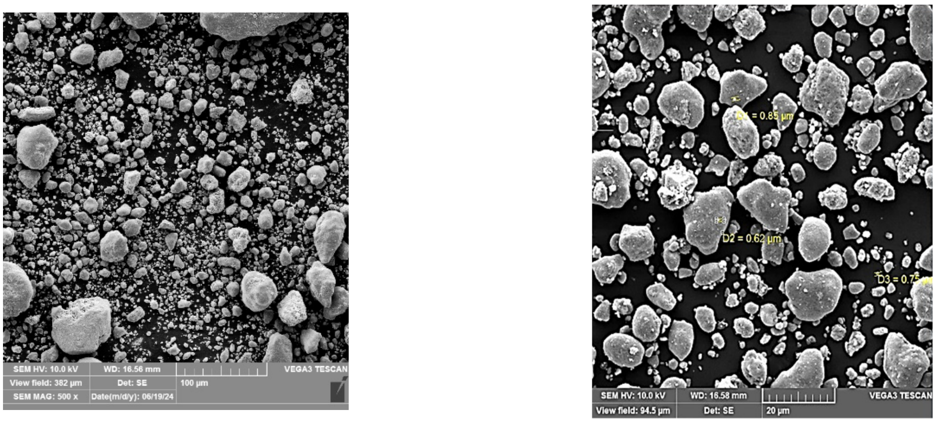

Figure S1. Morphology of zinc oxide

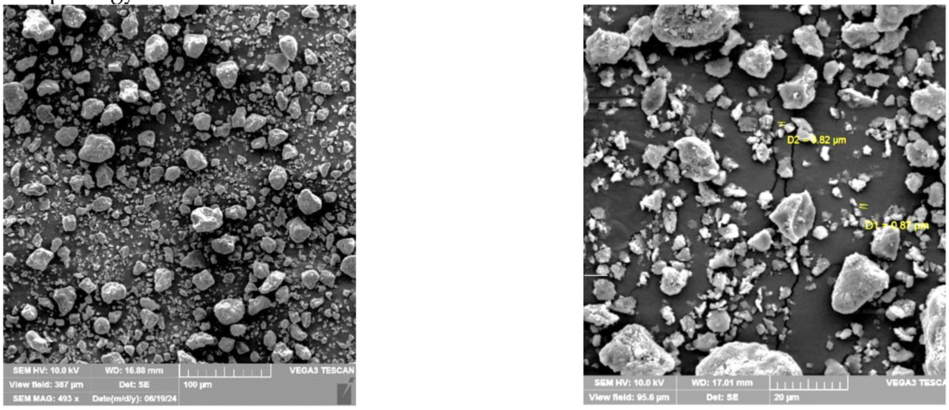

Figure S2. Morphology of zinc stearate

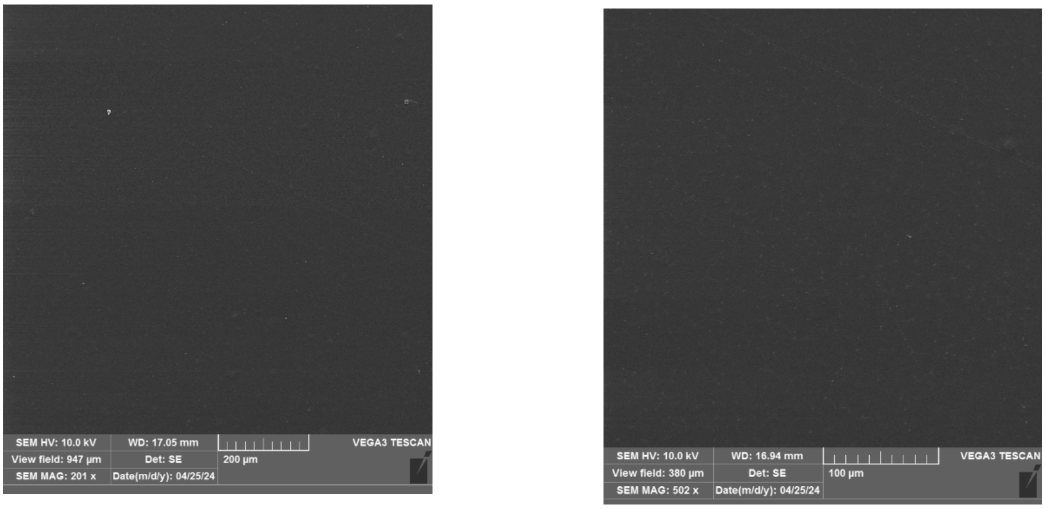

Figure S3. Morphology of PLA film

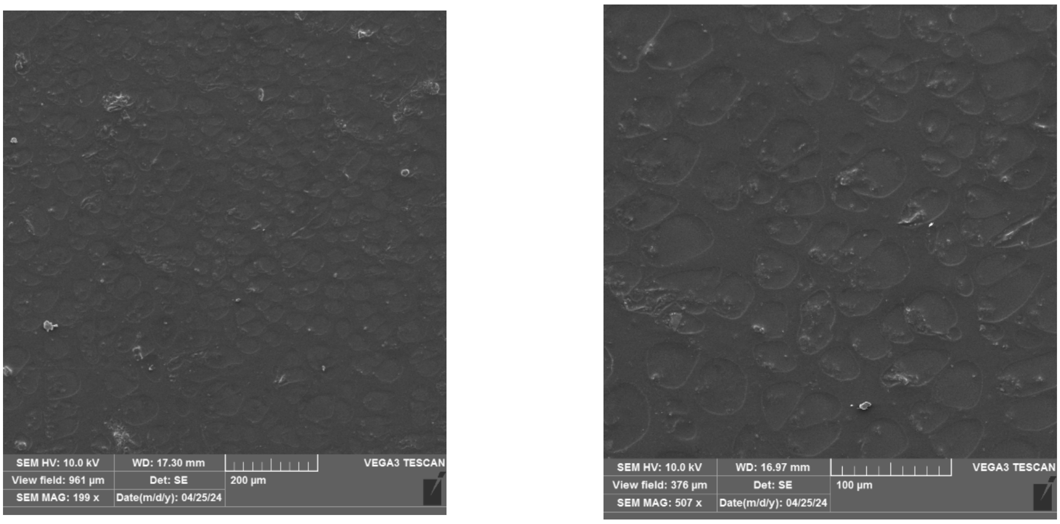

Figure S4. Morphology of PLA covered with HPMC coating carrier with emulsifier

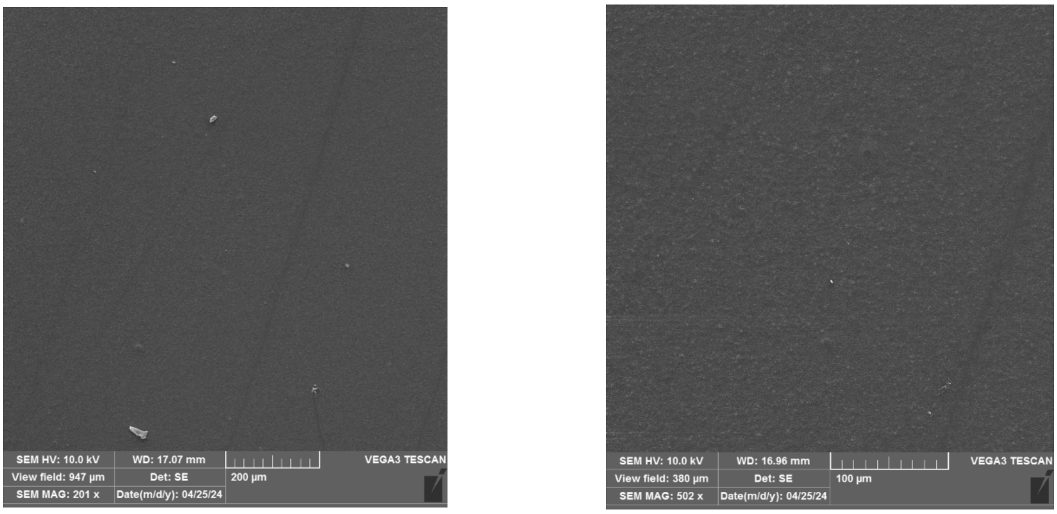

Figure S5. Morphology of PLA covered with EC coating carrier.

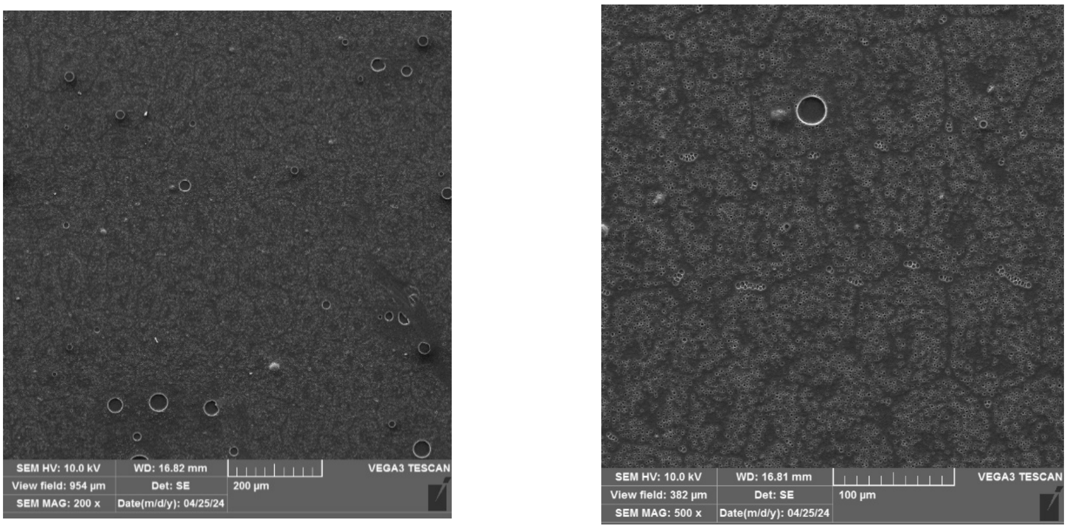

Figure S6. Morphology of PLA covered with AC1 coating.

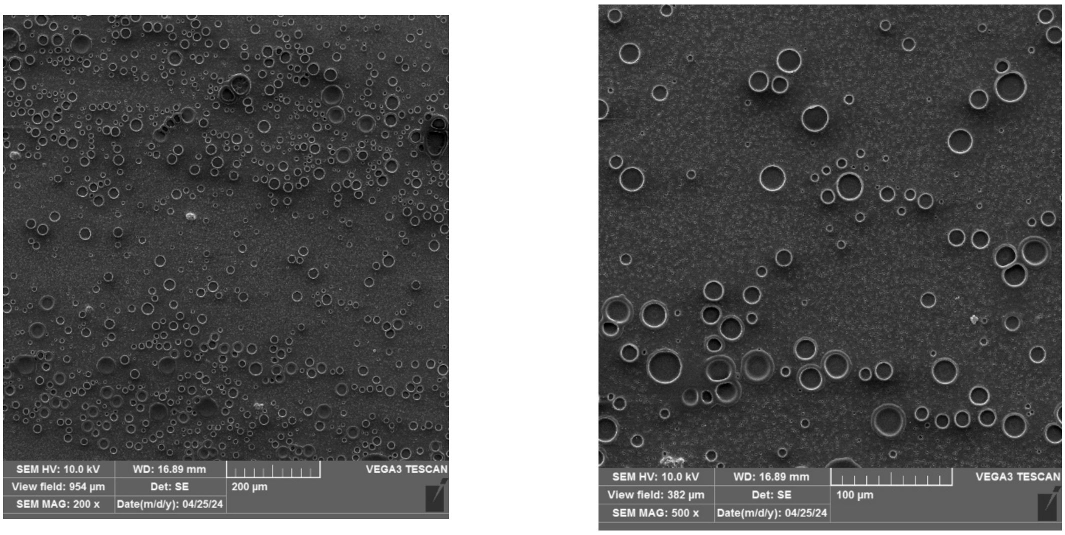

Figure S7. Morphology of PLA covered with AC2 coating.

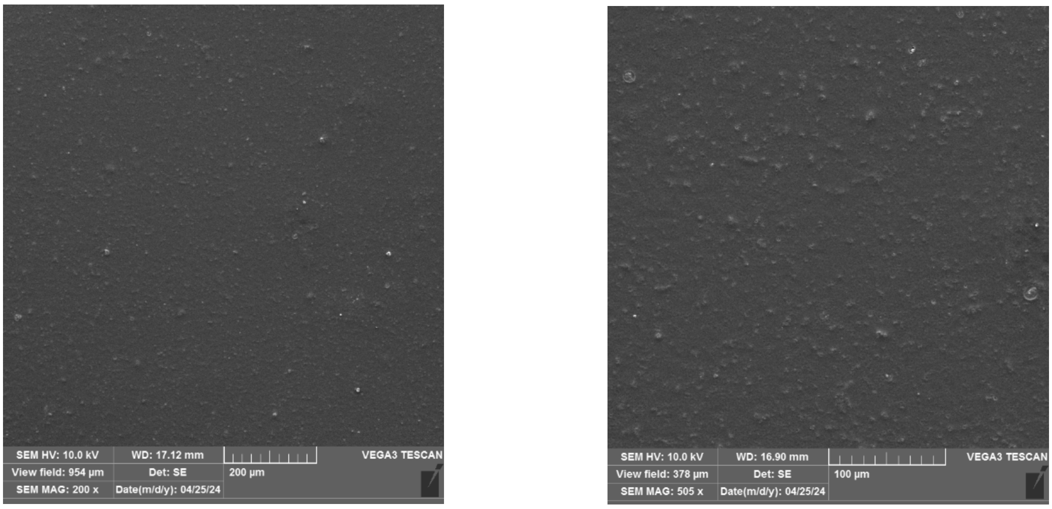

Figure S8. Morphology of PLA covered with AC3 coating.
